# Development of a fully automated workstation for conducting routine SABRE hyperpolarization

**DOI:** 10.1038/s41598-024-71354-x

**Published:** 2024-09-09

**Authors:** Jing Yang, Ruodong Xin, Sören Lehmkuhl, Jan G. Korvink, Jürgen J. Brandner

**Affiliations:** 1 Karlsruhe Institute of Technology (KIT), Institute of Microstructure Technology (IMT), 76344 Eggenstein-Leopoldshafen, Germany; 2 Karlsruhe Nano Micro Facility (KNMFi), Hermann-von-Helmholtz-Platz 1, 76344 Eggenstein-Leopoldshafen, Germany

**Keywords:** Chemistry, Engineering

## Abstract

SABRE is emerging as a fast, simple and low-cost hyperpolarization method because of its ability to regenerate enhanced NMR signals. Generally, SABRE hyperpolarization has been performed predominantly manually, leading to variations in reproducibility and efficiency. Recent advances in SABRE include the development of automated shuttling systems to address previous inconsistencies. However, the operational complexity of such systems and the challenges of integration with existing workflows hinder their widespread adoption. This work presents a fully automated lab workstation based on a benchtop NMR spectrometer, specifically designed to facilitate SABRE of different nuclei across different polarization fields. We demonstrated the capability of this system through a series of routine SABRE experimental protocols, including consecutive SABRE hyperpolarization with high reproducibility (average standard deviation of 1.03%), optimization polarization of ^13^C nuclei respect to the polarization transfer field, and measurement of polarization buildup rate or decay time across a wide range of magnetic fields. Furthermore, we have iteratively optimized the durations for pulsed SABRE-SHEATH ^13^C pyruvate. The constructed SABRE workstation offers full automation, high reproducibility, and functional diversification, making it a practical tool for conducting routine SABRE hyperpolarization experiments. It provides a robust platform for high-throughput and reliable SABRE and X-SABRE hyperpolarization studies.

## Introduction

Nuclear Magnetic Resonance (NMR) is recognized as a powerful analytical technique, yet it is commonly hindered by low sensitivity. Among the hyperpolarization methods developed to address this issue, Signal Amplification By Reversible Exchange (SABRE), a subset of Parahydrogen Induced Polarization (PHIP), stands out as an straightforward and efficient method to enhance polarization by several orders of magnitude^1–3^. SABRE operates by transferring spin order from parahydrogen (*para*-$\text{H}_{2}$) to the substrate through reversible ligation to a metal-organic catalyst without chemically modifying the substrate, thereby allowing for the continuous regeneration of hyperpolarized (HP) species^4–6^.

SABRE and PHIP hyperpolarization can be implemented through three primary methods: shaking^7–9^, bubbling^4,10–12^, and utilizing a gas-liquid reactor (polarizer)^13–17^ . Among these, shaking exhibits the least reproducibility, largely attributable to variations in manual force, making it less suitable for automated processes^18,19^. The gas-liquid reactors and flow cell setups face challenges due to their large sample volumes and complex fluid management, hindering widespread adoption^15,20^. Additionally, achieving optimal hyperpolarization requires balancing between contact time and sample transfer time, often leading to compromises in polarization levels^6,21^. Bubbling is a simple method for delivering *para*-${\text{H}_2}$ into the sample solution, and it is compatible with field cycling as well as automation techniques. This makes it a preferred choice for enhancing reproducibility and scalability in SABRE applications^22–24^.

While some automated setups have demonstrated in specific SABRE application^15,23,25^ and shown improvements in the reproducibility of SABRE hyperpolarization. Cowley et al.^26^ integrated a reaction cell and a flow probe into an NMR spectrometer for automated hyperpolarization at a defined field . Tomhon et al.^22^ designed and constructed a pneumatic shuttling system compatible with the Bruker 400 MHz NMR spectrometer for fast field cycling . Alcicek et al.^23,25^ employed a robotic arm-assisted shuttling system to conduct relayed hyperpolarization and measured the signal at a benchtop NMR . Recently, Ellermann et al.^27^ developed a stepper motor-based shuttling system for ^1^H SABRE at a benchtop NMR. However, there is still room for enhancement, particularly in terms of achieving full automation and broadening functionalities for routine SABRE hyperpolarization. These routine SABRE protocols including activation of the pre-catalyst for SABRE hyperpolarization, field cycling for ^1^H via SABRE hyperpolarization in milliTesla (mT) range^28,29^ and heteronuclei, such as ^13^C, ^15^N via SABRE-SHEATH (signal amplification by reversible exchange-in shield enables alignment transfer to heteronuclei) at microTesla (μT) field^30–33^, and determining polarization buildup and relaxation times across various fields. Additionally, automated setups are ideal for studying the polarization mechanism, such as using field pulse sequences to enhance heteronuclei polarization under SABRE-SHEATH conditions^34–36^.

To address these challenges and take the opportunities, this study introduces a fully automated lab workstation for conducting routine SABRE and SABRE-SHEATH hyperpolarization at either mT or μT fields with high reproducibility through precise control over experimental conditions. Leveraging an integrated graphical user interface (GUI), even first-time users are able to conveniently operate various SABRE experimental protocols, making the automated workstation a practical tool for studying SABRE hyperpolarization.

## Methods

### Development of automated SABRE workstation

**Fig. 1:**
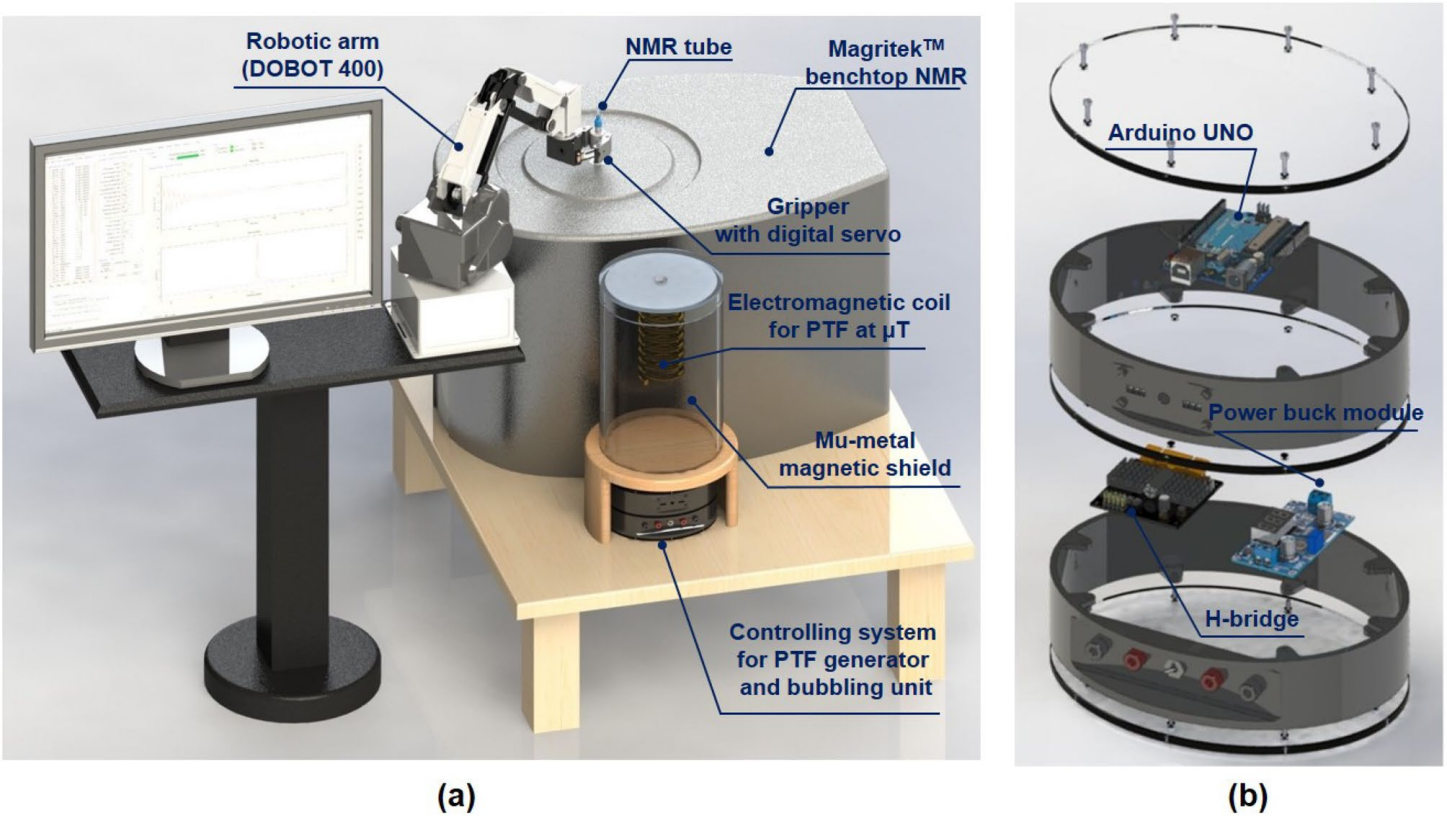
(**a**) 3D rendered overview of the automated SABRE workstation for benchtop NMR. The workstation consists of a robotic arm-assisted shuttling system, a self-wound solenoid coil placed in the mu-metal shielding as a μT-field PTF generator and a custom controlling system. (**b**) Explosion view of the controlling system. Several pins on the micro controller (Arduino® UNO R3) are configured to control the bubbling of *para*-${\text{H}_2}$ and the gripper on the robotic arm. With the PWM function of Arduino, the H-bridge driver is able to precisely generate the desired magnetic field on the electromagnetic coil.

The automated SABRE workstation integrates a commercial desktop robotic arm as a core component of the shuttling system for transferring the sample tube (Fig. 1a). The magnetic field for efficient polarization transfer with SABRE is generated by a self-wound solenoid coil positioned either inside or outside of a mu-metal magnetic shield. This component is referred to as the polarization transfer field (PTF) generator. A custom-built controlling system (Fig. 1b) controls the PTF generator, ensuring accurately manipulate both the strength and orientation of the magnetic field required for optimal hyperpolarization conditions. In the subsequent sections, each component of the system will be described in detail to provide a clear understanding of how they contribute to the overall experimental operation.

### Robotic arm-assist shuttling system

**Fig. 2:**
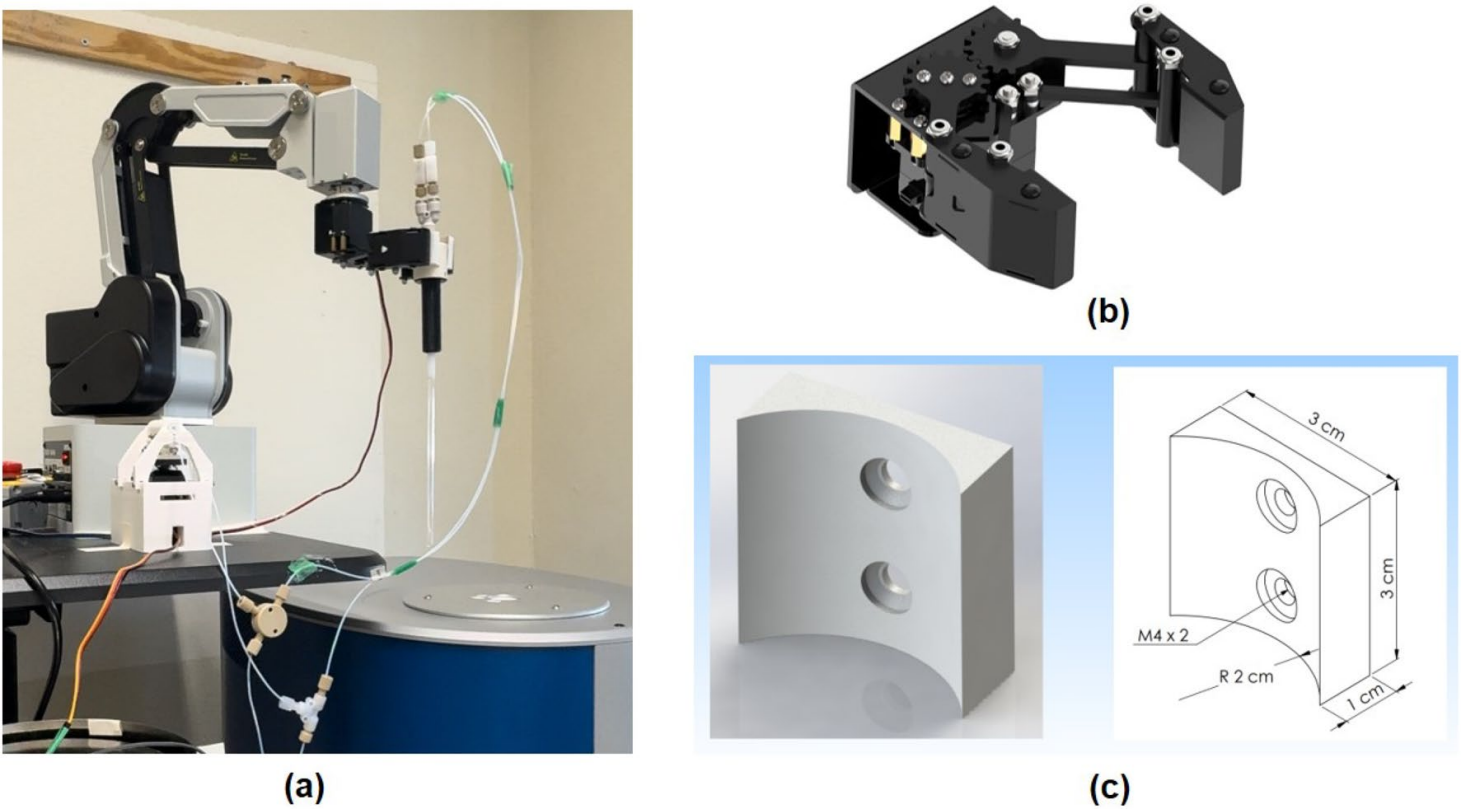
(**a**) The robotic arm-assisted shuttling system for fast magnetic fields cycling. (**b**) The anti-block gripper with high torque steering gear. The opening and closing of the gripper was independently controlled by a digital servo, allowing the action of grasping or releasing to be completed within 0.5 s. (**c**) 3D schematic of the finger for the gripper with a curved contour.

The 4-axis desktop robotic arm (Dobot MG400, Shenzhen, China) featuring a repeatability of $\pm 0.05$ mm and a maximum joint speed of $300^\circ$ per second, enables precise and rapid field cycling of the sample (Fig. 2a). The movement of the robotic arm, including the position coordinates and speed, can be customized through programming in a Python environment. Consequently, the desired position of sample tube can be programmed by inputting the corresponding spatial coordinates for the gripper (Fig. 2b). The gripper is equipped with a digital servo (LDX-335MG, Hiwonder, China) and features a custom 3D-printed finger designed to match the curvature of the sample holder (Fig. 2c). By programming the gripper to open or close, it is capable of seamlessly picking up and dropping off sample tubes at desired positions. In this work, the average speed of movement of the robotic arm was set to $70\%$ of its maximum capacity to balance the transfer speed with safety considerations. Consequently, the transfer time of the hyperpolarized (HP) sample from the PTF to the detection area of the benchtop NMR was consistently maintained at 3 s across all experiments.

### Bubbling unit

**Fig. 3:**
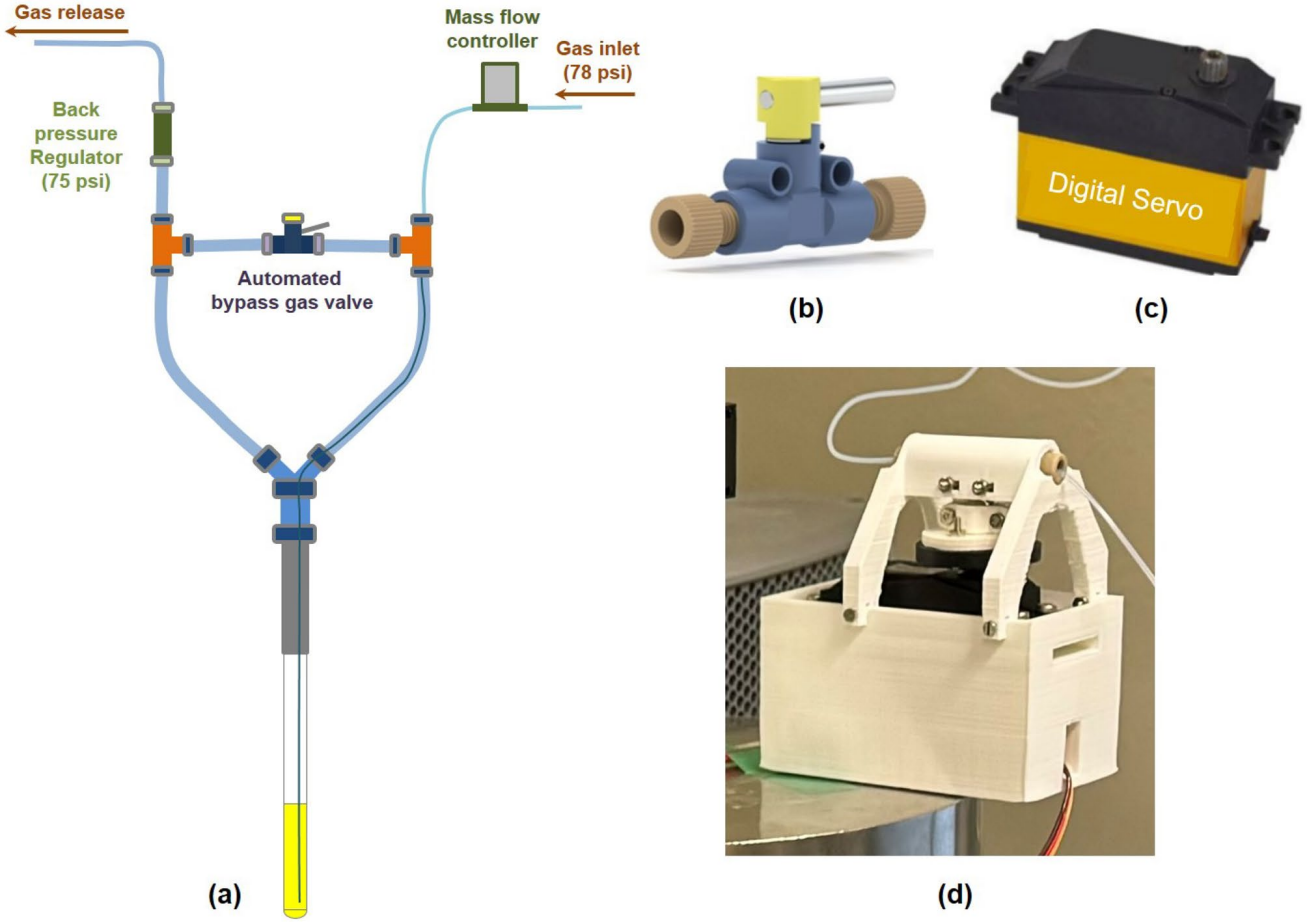
(**a**) Schematic drawing of the bubbling unit (not shown in real scale). The automated bypass gas valve includes a shut-off valve (**b**), a digital servomotor (**c**), and a scaffold (**d**) for mounting these components together. By controlling the digital servomotor, the automatic bubbling of *para*-${\text{H}_2}$ into the sample solution through the capillary tubing is managed.

As it mentioned in the introduction, bubbling method is compatible with fields cycling and automated setup. In this work, we built an automated bubbling unit for efficiently delivering *para*-${\text{H}_2}$ into the sample solution (Fig. 3).

The bubbling unit is comprised of a standard 5 mm NMR tube, to which PTFE tubing (ID: 4.35 mm, OD: 6.35 mm) is affixed at the upper portion. The other end of this PTFE tubing is connected to a Y-type fast pneumatic connector, splitting the gas flow into two pathways: one for bubbling and the other for gas release through a back-pressure regulator (BPR) with a cartridge of 75 psi (P-786, IDEX Health&Science, USA) mounted at the downstream (Fig. 3a). The source pressure of *para*-${\text{H}_2}$ in the gas bottle was initially at 30 bar. To adapt this pressure for the experimental requirements, a gas valve was employed to reduce it. The pressure was adjusted down to 78 psi at the inlet of the mass flow controller. The flow rate of *para*-${\text{H}_2}$ was controlled by a mass flow controller (MC-500SCCM-D, Alicat Scientific, USA). In the bubbling pathway, a capillary tubing (ID: 0.30 mm, OD: 0.60 mm) is inserted into the outer tubing, with its end submerged in the sample solution. A bypass line (PTFE tubing, OD: 3.2 mm, ID: 1.0 mm), created using two T-connectors, bridges the bubbling and releasing pathways to ensure equal pressure in both. A shut-off valve ( P-782, IDEX Health&Science, USA) controls the state of the bubbling. When this valve is open, bubbling ceases, and the gas is diverted through the bypass line and subsequently released. Closing the valve blocks the bypass, directing the gas through the capillary tubing where it forms bubbles in the sample solution, before being released through the BPR. This setup was previously introduced in earlier research, where the bubbling process was controlled by manually opening and closing the shut-off valve (Fig. 3b)^37^.

Throughout the experiment, the tubing of the bubbling unit remained attached to and moved in conjunction with the NMR tube. This configuration was integral to automating the process. However, it should be note that having the capillary immersed in the sample solution for bubbling could potentially broaden the linewidth of the NMR spectrum due to the additional magnetic field inhomogeneities introduced by the capillary’s presence within the sample.

To automate the bubbling procedure, traditional solenoid valves could be employed as replacements for the manual shut-off valve. However, when controlling hydrogen gas, the solenoid valve must be explosion-proof, which increases the cost and requires more installation space. To enhance experimental safety while maintaining automation, a digital servomotor (Amewi AMX Racing Digital Servo HV2060MG) is implemented to automatically drive the handle of the shut-off valve (Fig. 3c). A 3D-printed scaffold, as depicted in Fig. 3d, is utilized to assembly of the servomotor and the shut-off valve. The primary benefit of this mechatronic modification to the manual valve is the automated control of the hydrogen gas valve, achieved in a cost-effective and safe manner. Additionally, leveraging the rapid response capability of the digital servomotor, the bubbling process can be switched on and off by the digital servomotor within 0.5 s, ensuring consistent operation of the bubbling process and enhancing the reproducibility of experiments.

### PTF generator

SABRE possesses a well-known magnetic field dependence, for which an optimal PTF is essential to achieve spontaneous and efficient spin order transfer of *para*-${\text{H}_2}$ to the target substrates. In this study, we constructed two solenoid coils specifically designed for SABRE hyperpolarization of ^1^H, and heteronuclei, respectively. The first coil is positioned within the stray magnetic field of a benchtop NMR to access fields in the mT range, suitable for ^1^H SABRE experiments. The second coil, intended for heteronucleus hyperpolarization via SABRE-SHEATH, is capable of generating PTFs ranging from $-13.5$ to $13.5$ μT. This coil is housed within a mu-metal magnetic shield (ZG 206, Magnetic Shield Corporation, USA) to shield the Earth’s magnetic field.

**Fig. 4:**
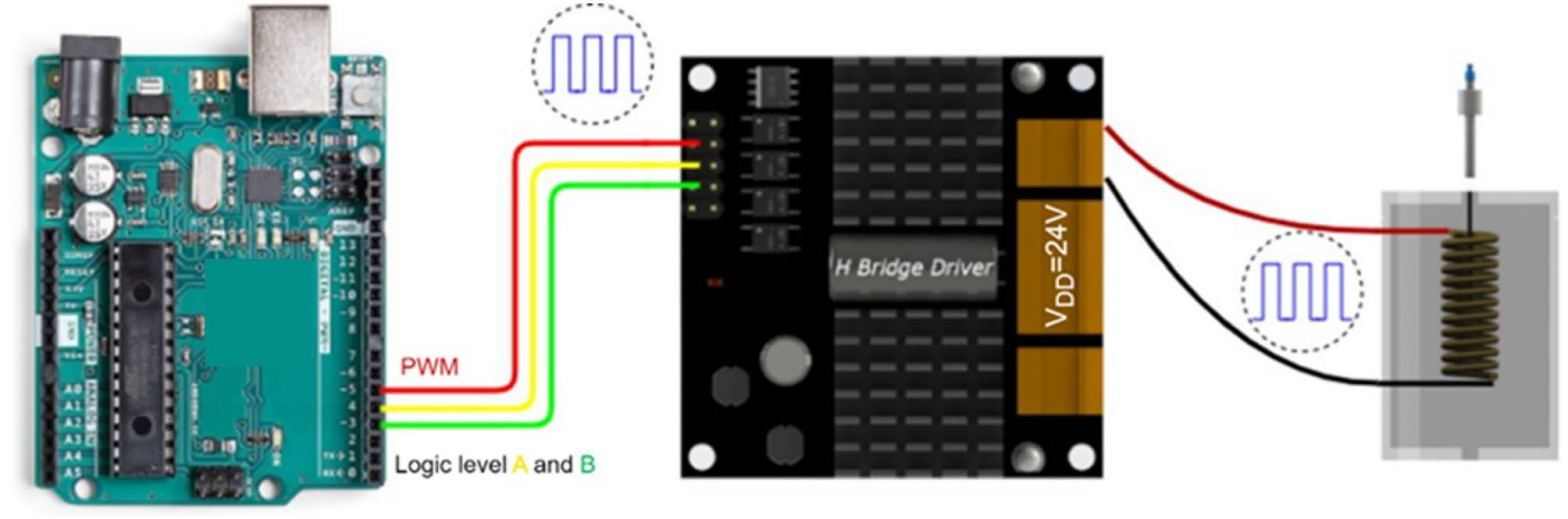
Schematic diagram of the circuit connections of the PTF generator. By combining the functions of an H-bridge and PWM on Arduino, it is capable to precisely control both strength and direction of the magnetic field generated on the solenoid coil.

The core component of the PTF generator is an H-bridge-based drive circuit that incorporates pulse width modulation (PWM). The H-bridge configuration consists of four switches, which can be transistors or relays, arranged in a manner that allows current to flow through the solenoid in either direction. By programming and toggling these switches in various combinations, it is possible to alter the direction of the magnetic field induced in the coil.

The schematic diagram of the circuit connections of the PTF generator is detailed in Fig. 4. The process for generating PTFs operates as follows: the micro controller (Arduino® UNO R3) is programmed to emit a square wave signal, which can be modulated using PWM to produce signals with varied duty cycles. Consequently, the H-bridge driver board then outputs an average voltage based on the square wave signal, generating the designated PTF within the electromagnetic coil. More details of the PTF generator can be found in Supporting Information. With a minimum duty cycle change of 1/255 of the full signal, the resolution of the PTF generator achieves a precision of 0.05 milligauss (mG). By programming the corresponding duty cycles into the micro controller, we were able to precisely and automatically sweep the PTF for each SABRE hyperpolarization to determine the optimal PTF for various substrates.

In addition, the PTF generator is capable of producing a field pulse sequence that consists of two alternating magnetic fields with different durations. This functionality facilitates investigation of the polarization transfer mechanisms in SABRE.

### User-friendly GUI

**Fig. 5:**
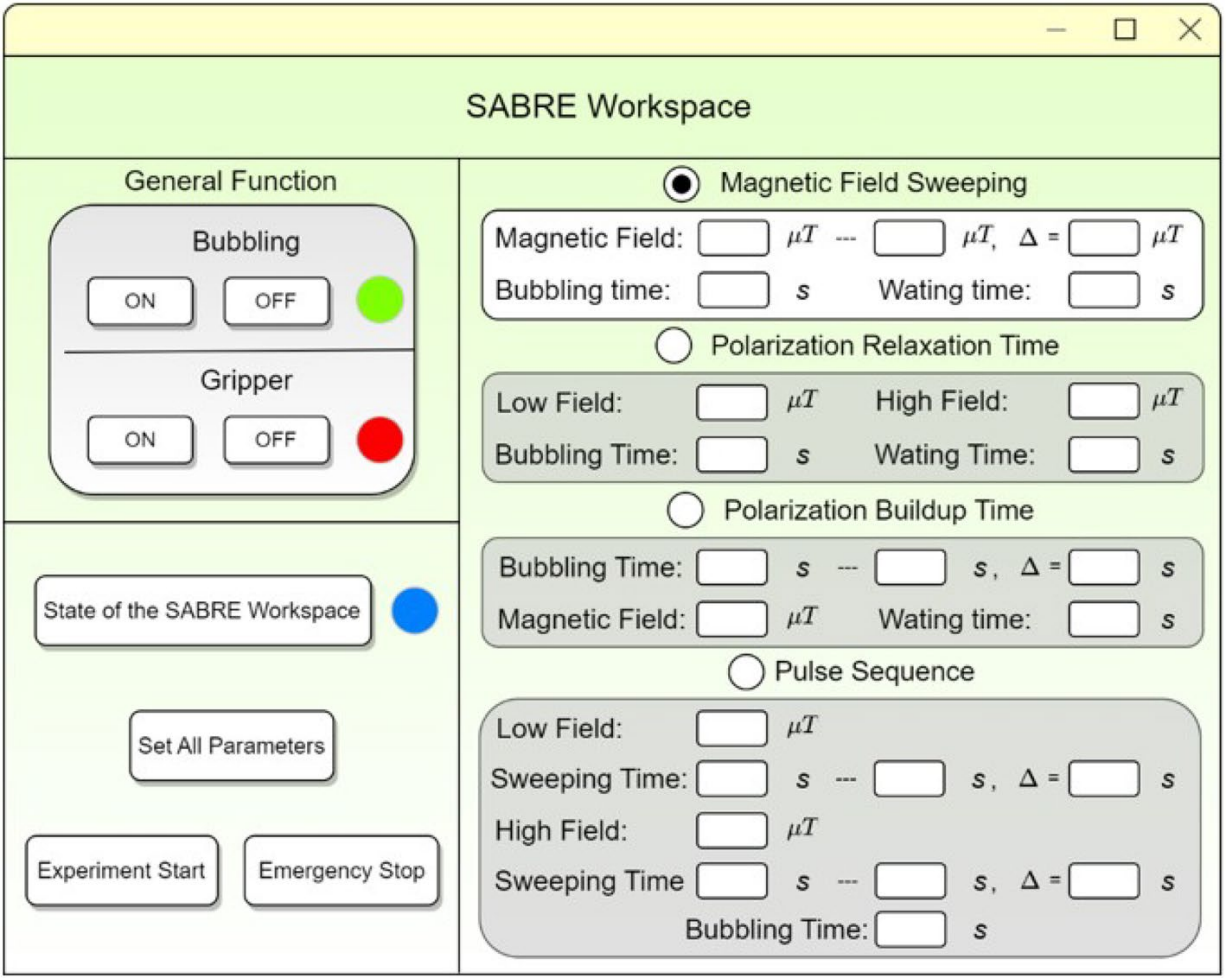
The main window of the GUI developed for controlling SABRE and SABRE-SHEATH experiments, showing the layout and functional elements. Upper left: general functions for activating the pre-catalyst by clicking the Bubbling On or OFF, and for grasping and releasing the sample tube by clicking the corresponding ON or OFF buttons. Bottom left: state indicator and issuance of instructions. Right column: four categories of SABRE experimental procedures, each of which can be selected by selecting the corresponding radio button.

A GUI was designed based on the experimental procedures of SABRE and SABRE-SHEATH, and allows users to easily control various parameters, such as the strength and direction of the PTF, the bubbling time, and configure the field pulse sequence. It includes features such as real-time monitoring of system status, automated sequence initiation, and data logging capabilities. The main window of this GUI is depicted in Fig. 5.

The upper left part of the GUI features the general function module, where users can individually operate the bubbling and gripper functions by clicking the corresponding buttons. This design allows for routine tasks such as activating the SABRE pre-catalyst in a sample solution, or moving the sample tube to a desired position. For instance, the sample tube can be transferred to the benchtop NMR for detection of the thermal equilibrium signal after activation. The real-time status of the SABRE workstation is visualized by different colors displayed at the bottom of the GUI: blue signifies standby, green indicates operating, and red alerts to a bug. Clicking the Experiment Start button without selecting any additional procedures or inputting parameters will automatically initiate the SABRE experiments using a set of default parameters. These default settings involve bubbling at the PTF of $+0.4$ μT for 40 s with a *para*-${\text{H}_2}$ gas flow rate of 85 sccm and a pressure of 90 psi (= 0.621 MPa). Immediately after bubbling, the sample is transferred to a benchtop NMR for detection. Additionally, all ongoing operations can be immediately halted by clicking the Emergency Stop button for ensuring the safety of both the personnel and the workstation in case of any unexpected situations. However, it is important to note that engaging this emergency stop will not result in the depressurization of the system.

Within the GUI, users can operate four categories of SABRE experimental procedures: magnetic field sweeping, measurements of polarization relaxation time, measurements of polarization buildup time, and pulse sequence. Users can select the desired experiments and input the necessary parameters displayed in each module to initiate the experiments. By clicking the Set All Parameters button, the parameters in GUI were written into a multi-threading python program. During the experiments, the program will execute the corresponding experimental procedure and call the API of SpinSolveExpert (prospa.exe) to execute the signal acquisition and saving the data. The subsequent sections will detail each procedure and present the corresponding experimental results to demonstrate the capabilities of the constructed system.

### Chemical sample preparation

#### Sample solution for ^1^H SABRE and ^13^C SABRE-SHEATH hyperpolarization

All SABRE sample solutions were prepared under inert gas conditions. For SABRE hyperpolarization of ^1^H substrates (pyrazine, pyridine, and nicotinamide), each sample solution contained 3 mmol/L of standard homogeneous SABRE pre-catalyst [IrCl (COD) (IMes)] (IMes=1,3-bis(2,4,6- trimethylphenyl)imidazole-2-ylidene; COD=cyclooctadiene)) and 60 mmol/L of each substrate dissolved in degassed methanol-d_4_.

For SABRE-SHEATH and pulsed SABRE-SHEATH of [1-^13^C] pyruvate, each sample contained 5 mmol/L pre-catalyst [IrCl(COD)(IMes)] (the same catalyst used in $^1$H SABRE), 25 mmol/L sodium [1-^13^C] pyruvate, and 40 mmol/L water-free DMSO dissolved in degassed methanol-d$_4$. All the chemicals were purchased from Sigma Aldrich and used without further purification.

#### Enrichment of *para*-${\text{H}_2}$

Normal hydrogen gas ($\hbox {ALPHAGAZ}^\text {TM}$ ${\text{H}_2}$, 99.999%) flows along the pipeline with an appropriate flow rate (0.5 mL/min) controlled by a Ex-proof mass flow controller (F-201CX / F-211CX, Bronkhorst, Germany). A commercial water-cooled helium compressor (Model ARS-4HW, Advanced Research System) offers the cooling source to the custom-tailored cryostat (DE-204AE 9K, Advanced Research System, USA). The enrichment process begins by cooling the cryostat to a stable temperature of 25 K. Subsequently, normal hydrogen gas is introduced into the catalyst chamber inside the cryostat. After contacting the paramagnetic hydrated iron(III) oxide catalyst (${Fe_{2}O_{3}\cdot H_{2}O}$) for facilitating the *o*${\text{H}_2}$-*p*${\text{H}_2}$ conversion, *para*-${\text{H}_2}$ with fraction of 98% was generated and collected within an aluminium gas bottle (DIN477-1, Knautz GmbH & Co. KG, Germany). More details about the *para*-${\text{H}_2}$ enrichment apparatus is comprehensively described by Hoevener et al.^38^ in their previous work.

#### Activation of the pre-catalyst

The prepared sample solution was deposited in the modified 5 mm NMR tube with a 10 cm long needle syringe and capped tightly until connected to the tubing of the bubbling unit. Before starting the experiments, the tubing path of the bubbling unit was flushed with fresh *para*-${\text{H}_2}$ for 2 minutes with a flow rate of 30 sccm at ambient pressure to ensure no residual air remained. After connecting the sample tube to the pneumatic connector of the bubbling unit, the flow rate was set to 85 sccm until the bubbling unit reached a stable over-pressure of 90 psi. Activation of the sample is initiated by clicking the ON button of the bubbling module in the GUI window. Full activation of the SABRE pre-catalyst typically requires 10 min of bubbling time.

### Experimental protocols

All the experiments conducted under the room temperature of $25\,^\circ$C. All NMR signals were measured on a Benchtop NMR operating at a ^1^H frequency of 61.92 MHz (1.45 T, Spinsolve 60 Carbon Ultra, Magritek, Germany). The ^1^H NMR spectra were obtained with a single pulse excitation experiment, collecting single scan of 32768 points with 200 μs dwell time over a sweep width of 20 ppm using $90^\circ$ RF pulse with length of 15.8 μs (signal acquisition time 6553.6 ms). The ^13^C NMR spectra were obtained with a single pulse excitation experiment, collecting single scan of 16384 points with 200 μs dwell time over a sweep width of 240 ppm using $90^\circ$ pulse length of 81.9 μs (signal acquisition time 3276.8 ms). All the recorded NMR data were processed using MNOVA Software (Mestrelab Research), where the acquired free induction decays (FIDs) were apodized with an exponential filter of 0.3 Hz.

#### Regeneration of ^1^H HP substrates with SABRE at 6.5 mT

**Fig. 6:**
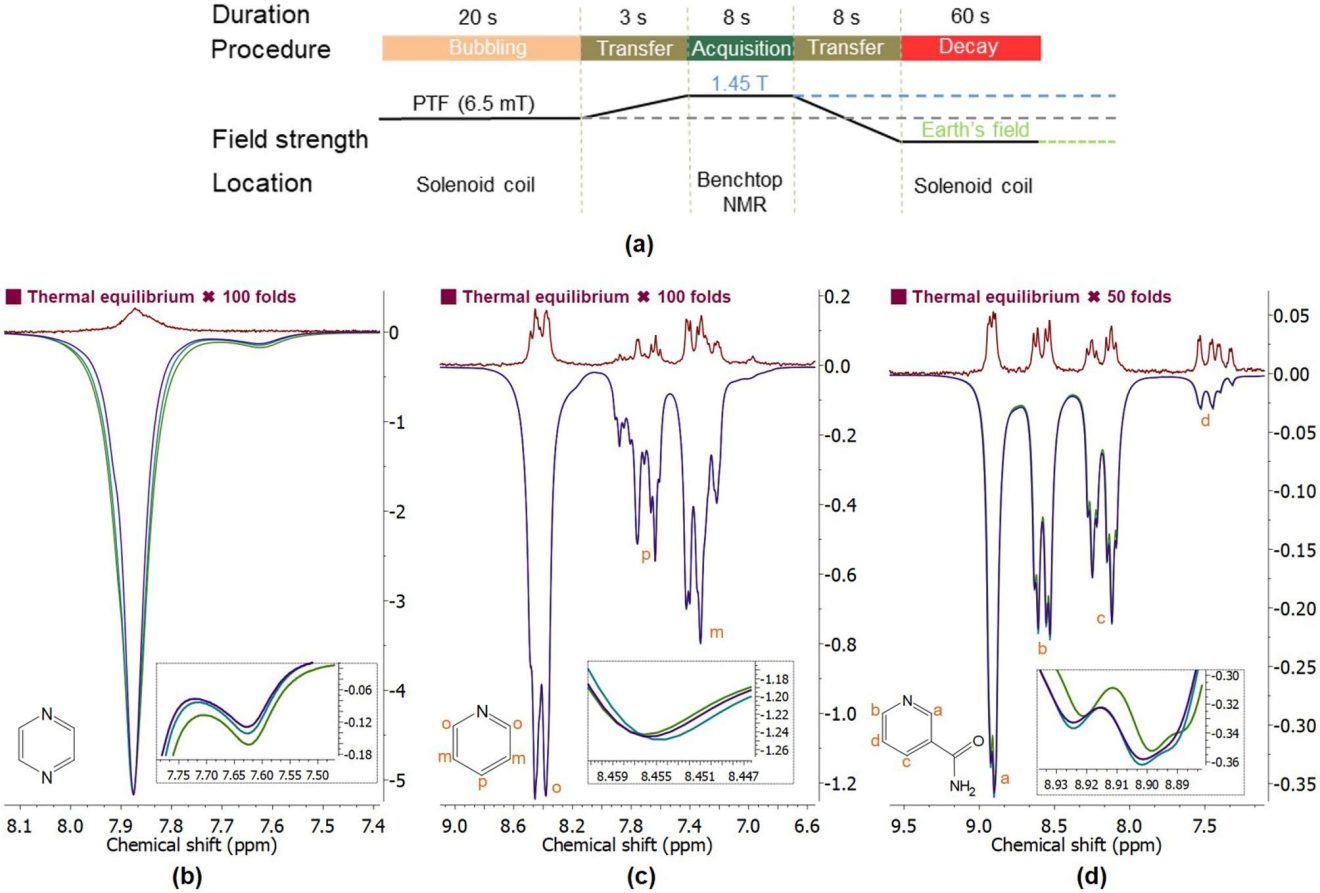
^1^H SABRE hyperpolarization of pyrazine, pyridine and nicotinamide executed by the automated SABRE workstation. (**a**) Schematic illustration of the experimental protocol for consecutive SABRE hyperpolarization at a PTF of 6.5 mT. (**b**–**d**) ^1^H spectra of the thermal references of each sample (red) and the corresponding HP pyrazine, pyridine and nicotinamide generated by repeating the protocols for three times. Insets: zoomed-in views of the HP spectra (in purple, green, and cyan) for each substrate.

^1^H SABRE hyperpolarization of each sample was performed in three consecutive trials using this automated workstation with the procedure shown in Fig. 6a. The activated sample was bubbled for 20 s with flow rate of 85 sccm at 90 psi at the PTF of 6.5 mT. Immediately after this, the samples were transferred with the robotic arm-assisted shuttling to the benchtop NMR within 3 s to measure the hyperpolarized ^1^H signal. After each measurement cycle, the sample was transferred back into the solenoid coil with the power off for 60 s. During this period, the signal relaxed back to its thermal equilibrium at Earth’s magnetic field. This process readied the sample for the next batch of hyperpolarization, ensuring that each cycle started from a consistent baseline state.

#### Studying field dependency in SABRE-SHEATH of [1-^13^C] pyruvate

**Fig. 7:**
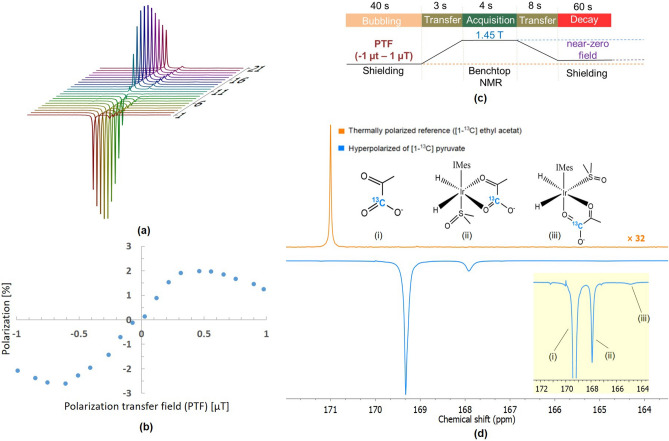
Magnetic field dependency on polarization level of [1-^13^C] pyruvate with SABRE-SHEATH. (**a**) Stacked NMR spectra of HP [1-^13^C] pyruvate hyperpolarized with SABRE-SHEATH at various PTFs sweeping from − 1 to 1 μT. (**b**) Polarization levels of the HP free pyruvate at corresponding PTFs. (**c**) Schematic illustration of experimental procedure for studying field dependency. (**d**) NMR spectra of the thermal reference sample of [1-^13^C] vinyl acetate (orange, signal intensity was enlarged with 32 folds) and [1-^13^C] pyruvate hyperpolarized at − 0.6 μT (blue) shows in free (i) and different bound forms (ii and iii). The polarization level of the free [1-^13^C] pyruvate is 2.6%.

The experimental protocol of ’Magnetic Field Sweeping’ on the GUI window was chosen for studying field dependency in SABRE-SHEATH (Fig. 5). As the procedure illustrated in Fig. 7c, the activated [1-^13^C] pyruvate sample was hyperpolarized at different magnetic fields by sweeping the generated PTFs in increments of $\Delta B_{\text {PTF}} = 0.1$ μT from − 1 to 1 μT. In each cycle, the sample solution was bubbled for 40 s with a flow rate of 85 sccm at 90 psi inside the degaussed magnetic shield at the desired PTF. Upon immediate cessation of the *para*-${\text{H}_2}$ bubbling, the sample was transferred to the benchtop NMR for ^13^C detection. After each acquisition, the sample was transferred back into the magnetic shield with the solenoid coil powered off for 60 s. The ^13^C polarization then relaxed at near-zero field conditions back to its thermal equilibrium.

#### Measuring ^13^C polarization buildup time $T_B$ at optimal PTF

**Fig. 8:**
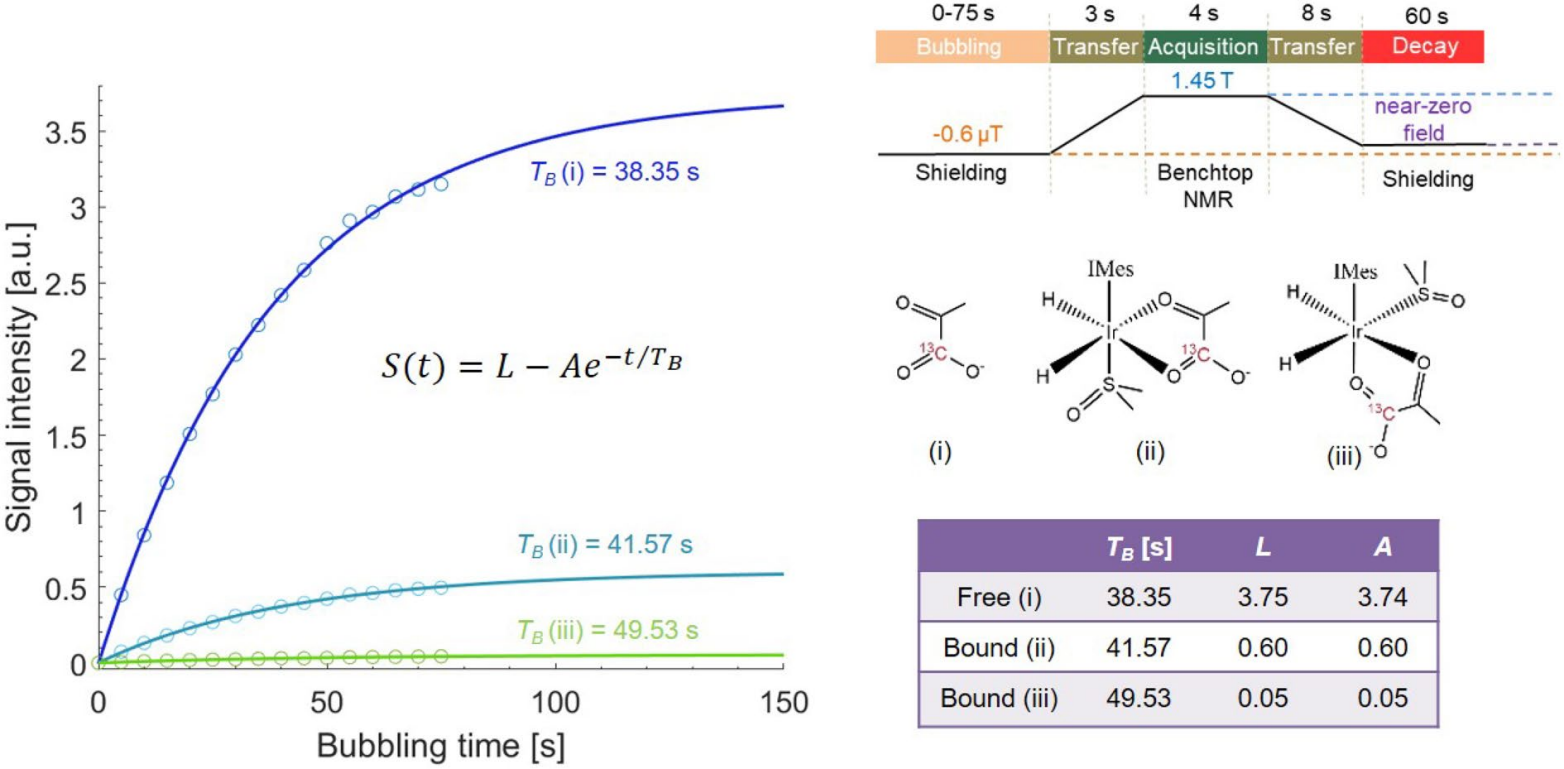
Measuring ^13^C polarization buildup time $T_B$ of free and bound pyruvate. Left: the measured polarization levels (circles) of the free pyruvate (i) and bound pyruvate (ii and iii) achieved with different bubbling times of *para*-${\text{H}_2}$ and the fitting curves with the model of a limited exponential growth equation $S(t) = L - A e^{-{t}/{T_B}}$. The parameter *L* is the limitation of the polarization level with infinite bubbling time *t* and *A* is initial difference from the limiting value. Right top: the schematic illustration of the experimental procedure for measuring the polarization buildup time. Right middle: chemical structures of the free and bound [1-^13^C] pyruvate. Right bottom: fitting results of polarization buildup time for the three types of pyruvate. For the pyruvate in forms of i, ii and iii, the corresponding $T_B$ are 38.35 s, 41.57 s and 49.53 s respectively.

The experimental protocol for ’Polarization Buildup Time’ was selected on the GUI window (Fig. 5). As the procedure illustrated in Fig. 8, the activated [1-^13^C] pyruvate sample was bubbled for varying durations (from 0 to 75 s in increments of 5 s) at its optimal PTF of − 0.6 μT and at $25\,^\circ$C. The sample was transferred to the benchtop NMR for detection immediately after cessation of bubbling. After each acquisition, the sample was transferred back into the magnetic shield with the solenoid coil powered off for 60 s. The ^13^C polarization then relaxed at near-zero field conditions back to its thermal equilibrium.

#### Measuring ^13^C polarization relaxation time $T_1$ at different interesting fields

**Fig. 9:**
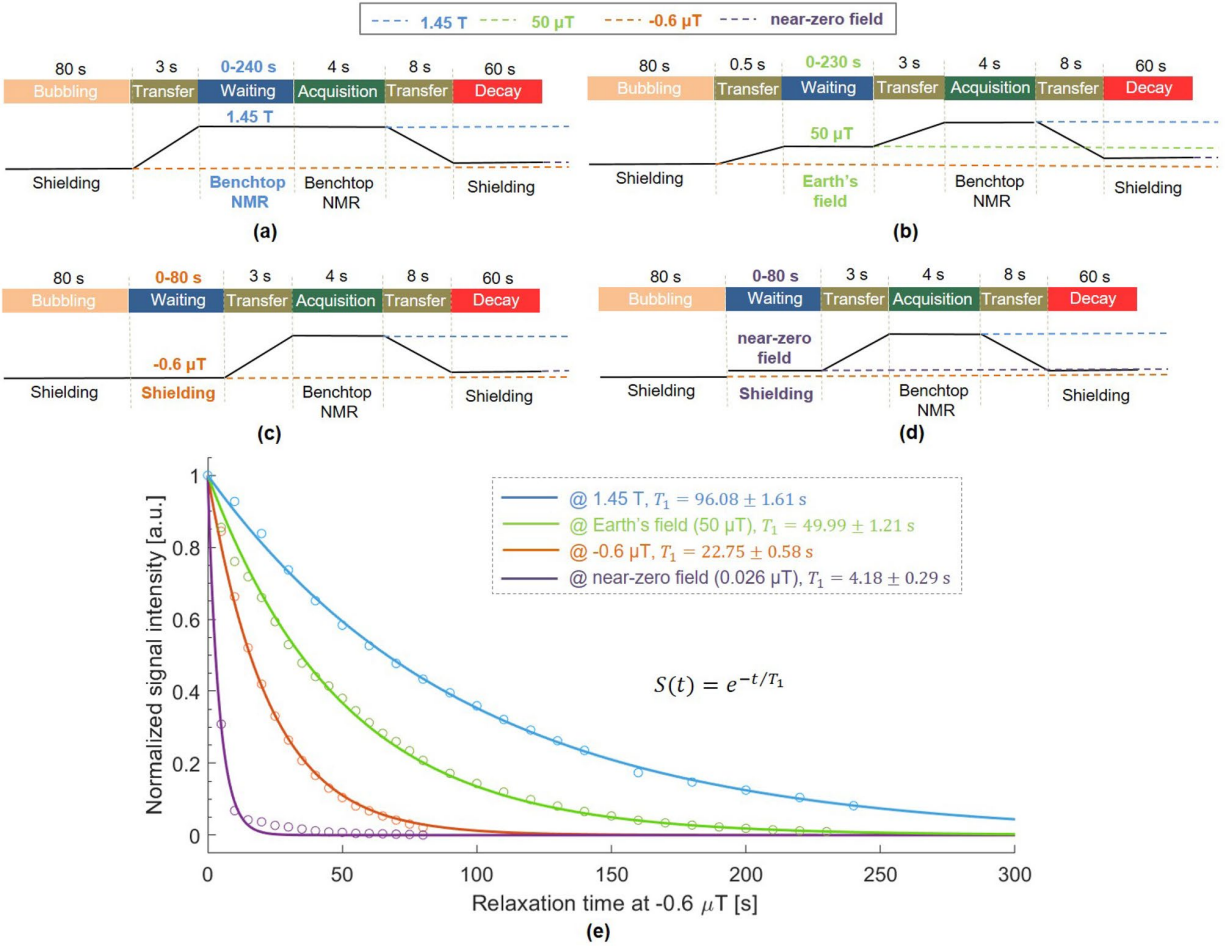
Measuring ^13^C polarization relaxation time of HP free pyruvate at four interesting magnetic fields. (**a**–**d**) Schematic illustration of experimental procedures for measuring polarization decay time of HP free [1-^13^C] pyruvate at 1.45 T, Earth’s field (50 μT), − 0.6 μT and near-zero field (0.026 μT). (**e**) Normalized signal intensity (circles) of HP free [1-^13^C] pyruvate relaxing under the four interesting fields. The polarization relaxation time $T_1$ was calculated by fitting the normalized signal intensity data to a mono-exponential equation $S(t) = e^{-{t}/{T_1}}$. The fitting results of the polarization relaxation time $T_1$ are 96.08 s at 1.45 T, 49.99 s at Earth’s field, 22.75 s at − 0.6 μT, and 4.18 s at near-zero field.

The experimental protocol for ’Polarization Relaxation Time’ was selected on the GUI window (Fig. 5). As the procedures illustrated in Fig. 9a–d, the sample was initially fully polarized by bubbling *para*-${\text{H}_2}$ at − 0.6 μT and $25\,^\circ$C for 80 s. Subsequently, the sample tube was immediately exposed to various magnetic fields to observe polarization relaxation. The sample was maintained inside the shield at − 0.6 μT, or kept near-zero field (0.026 μT) in situ, or moved outside the magnetic shield to Earth’s field (50 μT), or transferred to a benchtop NMR system at 1.45 T. After varying wait durations at these fields, ^13^C NMR signals were measured at the benchtop NMR. After each acquisition, the sample was transferred back into the magnetic shield with the solenoid coil powered off for 60 s. The ^13^C polarization then relaxed at near-zero field conditions back to its thermal equilibrium.

#### Pulse sequence of ^13^C SABRE-SHEATH hyperpolarization

The experimental protocol for ’Pulse Sequence’ was selected on the GUI window (Fig. 5). As the procedure illustrated in Fig. 11a, the sample was bubbled at a flow rate of 85 sccm and 90 psi at the set PTF with field pulse sequence, involving two alternating magnetic fields with corresponding durations $\tau _L$ and $\tau _H$ for each field. The low and high fields of the pulse sequence were set to near-zero field (0.026 μT) and 13 μT, respectively. Upon immediate cessation of the *para*-${\text{H}_2}$ bubbling, the sample was transferred to the benchtop NMR for ^13^C detection. After each acquisition, the sample was transferred back into the magnetic shield with the solenoid coil powered off for 60 s. The ^13^C polarization then relaxed at near-zero field conditions back to its thermal equilibrium..

## Results and discussion

### Estimating the reproducibility by regeneration of HP substrates with ^1^H SABRE at 6.5 mT

We conducted multiple times ^1^H SABRE hyperpolarization experiments on pyridine, pyrazine, and nicotinamide to evaluate the reproducibility of the constructed automated workstation. The enlarged thermal equilibrium spectra (shown in red) and the HP spectra (represented in purple, green, and cyan) are displayed in Fig. 6b–d.

The signal enhancements $\epsilon$ of the HP proton species on each substrates with three consecutive SABRE hyperpolarization at a PTF of 6.5 mT shown in Table 1. The reproducibility of SABRE hyperpolarization for each substrate was quantified by calculating the relative standard deviation (RSD) of $\epsilon$ observed for individual HP species. The results are presented in Table 1 with an average relative standard deviation $\overline{\epsilon }$ of $1.03\%$, indicating an improvement in reproducibility compared to other automated shuttling systems reported by other research groups. For instance, the pneumatic shuttling system operating at high-field conditions documented a standard deviation of 2%^22^, while the shuttling system employing a stepper motor and gear rod reported deviations ranging from 0.2 to 3%^27^.

**Table 1:** The calculated signal intensity enhancements $\epsilon$ of HP pyrazine, pyridine (across three sites: ortho, para, and meta), and nicotinamide (across four sites: a, b, c, and d).

	Pyrazine	Pyridine			Nicotinamide			
		ortho	para	meta	a	b	c	d
$\epsilon$ **1**	− 1494.92	− 1002.87	− 863.95	− 622.42	− 403.80	− 337.25	− 304.52	− 52.41
$\epsilon$ **2**	− 1528.72	− 1007.73	− 868.30	− 627.85	− 418.58	− 346.45	− 317.17	− 51.43
$\epsilon$ **3**	− 1466.27	− 1005.73	− 867.05	− 623.2	− 417.55	− 345.24	− 319.01	− 51.56
$\overline{P}_{{1}_{H}}$	0.31%	0.21%	0.18%	0.13%	0.09%	0.07%	0.07%	0.01%
RSD of $\epsilon$	1.71%	0.20%	0.21%	0.38%	1.63%	1.19%	2.05%	0.84%

The RSDs of the enhancements of each proton species on the three substrates are in range from 0.20% to 2.05%.

### Studying field dependency on polarisation transfer efficiency of HP [1-^13^C] pyruvate via SABRE-SHEATH

In this work, we studied the impact of magnetic field on the polarization transfer efficiency of [1-^13^C] pyruvate through systematically varying the magnetic field strength across a range of values. Previous works on simulations of the spin system at the Level Anti-Crossing (LAC) condition provides a reasonable estimation of the optimal PTF at 0.4 μT for [1-^13^C] pyruvate. However, the accuracy of the predictions are constrained by the simplification of the actual spin system dynamics, neglecting the influence of external parameters such as binding rates, dissociation rates, and fluctuations in real-time temperature^34,39–41^.

To determine the optimal PTF for [1-^13^C] pyruvate under conditions of this work, the PTFs were set in range from − 1 to 1 μT. As shown in Fig. 7**a**, the 21 NMR spectra of HP [1-^13^C] pyruvate with SABRE-SHEATH at various PTFs demonstrate a significant dependence of pyruvate signal intensities on both the strength and direction of the magnetic field. The variations in polarization level across different PTFs present an asymmetrical curve-like pattern (Fig. 7b). Notably, minor differences in the polarization levels at the same magnetic strength but in opposite directions (positive and negative) were observed.

Given that the Earth’s magnetic field in the z-direction measured in the laboratory is negative, the reduced signal intensity observed with positive PTFs was speculated due to alterations in the z-direction of the magnetic field when the sample is adiabatically removed from the shield and transferred to the benchtop NMR. The maximum signal intensity of free pyruvate with SABRE-SHEATH at $25\,^\circ$C is observed at − 0.6 μT (Fig. 7b). By taking a sample of [1-^13^C] vinyl acetate (in a normal 5 mm NMR tube) at thermal equilibrium as a reference (Fig. 7d), the calculated signal enhancement of HP free pyruvate is

1 $$\begin{aligned} \epsilon _{{{13}_\text{C}}} = \frac{S_{HP}}{S_{Ref}}\cdot \frac{C_{Ref}}{C_{Ref}} \cdot \frac{A_{Ref}}{A_{HP}} = \frac{13.1605}{0.2696} \cdot \frac{10.13 \, \text {mmol/L}}{25 \, \text {mmol/L}}\cdot 1.053 = 20828 \end{aligned}$$

The ^13^C thermal polarization level is

2 $$\begin{aligned} P_{\text {thermal}} = \frac{\gamma B_0 \hbar }{2kT} = \frac{67.262 \, \text {MHz/T} \cdot 1.45 \text {T} \cdot 1.0545718 \times 10^{-34} \, \text {Js}}{2 \cdot 1.380649 \times 10^{-23} \, \text {J/K} \cdot 298 \, \text {K}} = 1.25 \times 10^{-4} \% \end{aligned}$$

Therefore, the polarization level of HP free [1-^13^C] pyruvate with SABRE-SHEATH at − 0.6 μT and $25\,^\circ$C is

3 $$\begin{aligned} P_{{{13}_\text{C}}} = \epsilon _{{{13}_\text{C}}} \cdot P_{\text {thermal}} = 20808 \cdot 1.25 \times 10^{-4} \% = 2.6\% \end{aligned}$$

Higher polarization levels can be achieved using the temperature cycling method, which involves ice water cooling or air cooling the sample to $0\,^\circ$C during the bubbling process^42^. However, the cooling also introduce practical challenges, such as condensation forming on the exterior of the NMR tube. This condensation requires manual intervention, such as wiping the tube with a tissue before insertion into the NMR for detection. This additional step conflicts with the goals of full automation and continuous operation, rendering such cooling techniques currently incompatible with fully automated systems. Addressing this limitation would require modifications to the system design, perhaps by integrating an automated mechanism to manage or prevent condensation without manual intervention.

### Measuring ^13^C polarization buildup time $T_B$ at optimal PTF

The polarization build-up time in SABRE is influenced by several factors, including the chemical exchange rate within the substrate, the *J*-coupling network within the spin system, the strength of the PTF, the efficiency of *para*-${\text{H}_2}$ delivery to the sample solution, the sample transfer passage and the concentration of the chemicals. Each of these elements plays a role in determining how quickly and effectively polarization can be built up and maintained, affecting the overall polarization level achieved with SABRE.

We conducted the measurement of polarization buildup time $T_B$ of $[\text {1-}^{13}\text {C}]$ pyruvate at the optimal PTF of − 0.6 μT by varying the bubbling time. The results of the calculated polarization buildup times for both the free and catalyst-bound forms of pyruvate are illustrated in Fig. 8. The fitting curves were derived using a limited exponential growth equation to model the process of polarization development. The calculated polarization buildup time for the three forms of $[\text {1}-{^{13}\text{C}}]$ pyruvate (i, ii and iii) were 38.35 s, 41.57 s and 49.53 s respectively. This outcome, especially $T_B$ of the two bound form pyruvate (ii and iii), differs from previously reported results of ^13^C-pyruvate hyperpolarization at low temperatures, where the polarization buildup times for the two forms of catalyst-bound pyruvate are significantly shorter^42^. The primary reason for this discrepancy is that at low temperatures, the exchange of *para*-${\text{H}_2}$ is much more efficient than the exchange of pyruvate, which promotes rapid accumulation of polarization on catalyst-bound species^30,42,43^, leading to shorter polarization time compared to the condition at higher temperature.

### Measuring ^13^C polarization relaxation time $T_1$ of free pyruvate at different magnetic fields

As a crucial intermediate in numerous biochemical pathways, ^13^C pyruvate is an ideal marker to study and monitor various metabolic activities within biological systems^44^. A longer polarization relaxation time $T_1$ of ^13^C pyruvate allows a more extended monitoring period and observation of more metabolic products. $T_1$ relaxation time can be determined on an NMR spectrometer using an inversion recovery pulse sequence to measure the NMR signal over time. However, measuring the hyperpolarization relaxation time of HP substrates is challenging with this method, as they are typically generated at the PTF of low magnetic fields, where the spin-lattice relaxation may occur significantly faster.

In this work, we determined the ^13^C polarization relaxation time at different magnetic fields by using nuclear magnetic resonance dispersion (NMRD) measurements^45,46^. The HP sample was subjected to desired positions where the polarization allowed to decay at interesting fields. Four representative magnetic fields were selected for the experiments: a near-zero field, the optimal PTF of − 0.6 μT for ^13^C pyruvate, Earth’s magnetic field of approximately 50 μT, and the high field of 1.4 T from the benchtop NMR magnet. The decay at the first two fields smaller than Earth’s geomagnetic field is facilitated using the PTF generator within the shielding chamber. However, for the fields of 50 μT and 1.4 T, which exceed the capacity of the PTF generator (− 13.5 to 13.5 μT), the decay processes are practically conducted at the corresponding locations outside of the shielding chamber.

After decay at the desired magnetic field, the sample was then rapidly transferred to the benchtop NMR for measurement of the residual magnetization. This procedure was iterated multiple times, each with an extended decay duration at the same magnetic field. The measured signal intensities were then utilized to construct a relaxation curve for estimating $T_1$. The experimental procedures for measuring HP free ^13^C pyruvate relaxation time $T_1$ at 1.45 T, 50 μT, − 0.6 μT, and near-zero field are illustrated in Fig. 9a–d respectively. The blue block labeled “waiting” in the diagram indicates the intervals during which the HP sample was exposed to the corresponding magnetic fields for relaxation purposes.

The polarization decay curves of free pyruvate under different field conditions (circles) are shown in Fig. 9e. Each data point on the decay curves represents a periodic recording of the HP free pyruvate polarization using a $90^\circ$ RF pulse at the acquisition field of 1.45 T. A single exponential equation was used to fit the polarization relaxation curves at different fields. The analysis revealed that the polarization decay for free pyruvate varied significantly across different magnetic fields. Specifically, the relaxation times were measured as 96.08 s at 1.45 T, 49.99 s at the Earth’s magnetic field, 22.75 s at − 0.6 μT, and 4.18 s in a near-zero field environment.

### Optimization parameters of pulse sequence for ^13^C hyperpolarization

To demonstrate the diverse applications of the constructed workstation, we performed the pulsed SABRE-SHEATH experiments, an innovation technique in the field of SABRE hyperpolarization. This technique involves applying an oscillating pulse sequence to the electromagnetic coil during the bubbling phase, allowing the SABRE-SHEATH hyperpolarization to evolve at two alternating μT-fields, which are deviated from the optimal PTF. Despite these fields creating a weaker *J*-coupling network, they still facilitate the achievement of a high level of polarization. Preliminary results suggest that this capability stems from the fact that the heteronuclei coupling can be readily interconverted between strong and weak coupling regimes^36,43^. Therefore, pulsed SABRE-SHEATH allows investigating the complex spin dynamics and optimizing the generated polarization by varying the parameters of the pulse sequence.

**Fig. 10:**
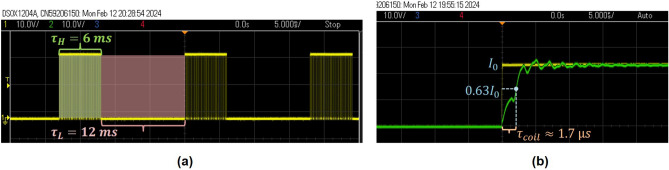
Left: example of a square wave signal consisting of two high-and low-levels with duration ($\tau _H = 6$ ms and $\tau _L = 12$ ms) applied to solenoid coil for generating two alternative fields. Right: Estimating the switching time $\tau _{coil}$ of the two magnetic fields verified on the oscilloscope (KEYSIGHT DSOX1240A).

We optimized ^13^C polarization by adjusting the parameters of the applied field pulse sequence. In this procedure, the two alternating magnetic fields $B_{\text {Low}} \approx 0$ μT and $B_{\text {High}} = 13$ μT with respective durations $\tau _L$ and $\tau _H$ were generated using a square wave consisting of two voltage levels (Fig. 10a). These voltage levels were modulated by PWM with corresponding duty cycles to precisely control the timing and intensity of each magnetic field. The switch time $\tau _{coil} = 1.7$ μs between high and low levels was determined by measuring the time it takes the system to reach approximately 63% of its stable state (Fig. 10b).

**Fig. 11:**
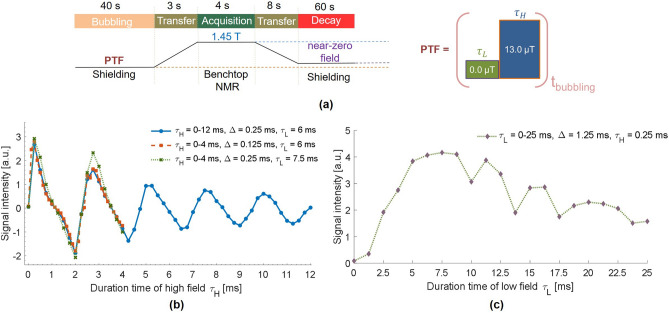
Optimizing the polarization level of free pyruvate with respect to durations in pulsed SABRE-SHEATH experiments. (**a**) Schematic illustration of the experimental procedure for pulsed SABRE-SHEATH. The sample solution was bubbled for 40 s at the PTF involving two alternating fields with corresponding durations of $\tau _L$ and $\tau _H$. (**b**) Signal intensities of HP free pyruvate hyperpolarized with sweeping the duration time $\tau _H$ in the high magnetic field (13 μT). (**c**) Signal intensities of HP free pyruvate hyperpolarized with sweeping the duration time $\tau _L$ in the low magnetic field (near-zero field). The maximum signal intensities occur when $\tau _H = 0.25 \, \text {ms}$ and $\tau _L = 7.5 \, \text {ms}$ of the pulse sequence. Lines connecting each measured point are included to guide the eyes.

To determine the optimum duration time of the low and high fields respectively, four series of pulsed SABRE-SHEATH of [1-^13^C] pyruvate were performed. Initially, we investigated the dependency of polarization on the duration of the high-field pulse while keeping the duration of the low-field pulse fixed at $\tau _L = 6\, \text {ms}$. By sweeping the duration of the high-field from 0 to 12 ms in increments of 0.25 ms, we observed that the signal intensity of the HP free pyruvate exhibited a curve resembling a sinusoidal (Fig. 11b, blue). As $\tau _L$ increases, the extremum of the polarization within the periodic interval gradually decreases. An additional set of experiments was conducted by sweeping $\tau _L$ from 0 to 4 ms with even smaller increments of 0.125 ms to more precisely determine the optimal duration of the high-field pulse. The results indicated that the maximum signal was observed when $\tau _L$ was set at 0.25 ms (Fig. 11b, orange). Building on this, we iteratively studied the dependency of polarization on the duration of the low-field pulse. By sweeping $\tau _L$ from 0 to 25 ms with an increment of 1.25 ms, the maximum polarization observed when $\tau _L$ was set at 7.5 ms (Fig. 11c). This is also demonstrated by the iterative experiments of $\tau _H$ sweeping with fixed $\tau _L = 7.5$ ms (Fig. 11b, green). Therefore, the optimal duration times of the high and low fields for pulsed SABRE-SHEATH were $\tau _H = 0.25$ ms and $\tau _L = 7.5$ ms, respectively.

## Conclusion

In summary, the constructed SABRE workstation with the robotic arm-assisted shuttling system and the controlling system allows fully automated execution of the SABRE and SABRE-SHEATH hyperpolarization experiments. The tailored GUI simplifies the implementation of various hyperpolarization protocols. These include sample activation, magnetic field sweeping, measurement of buildup and relaxation times under different field conditions, as well as the execution of pulsed SABRE-SHEATH experiments. The flexibility and automation of this setup have enabled multiple applications. These include continuous SABRE hyperpolarization of different substrates with high reproducibility and the precise determination of optimal experimental parameters, such as the polarization transfer field or pulse sequences. The automated SABRE workstation shows promise for optimizing polarization transfer efficiency, investigating the mechanisms of polarization transfer, and high-throughput testing the performance of new SABRE catalysts. In the future, we will also dedicate efforts to develop multi-channel hydrogen gas pipeline controls. This development will facilitate the integration and compatibility of the automated system with a multi-sample carousel, thereby meeting the demands for high-throughput hyperpolarization.

## Supplementary Information

Supplementary Information.

## Data Availability

The datasets generated and/or analyzed during the current study will be provided by the corresponding authors upon reasonable request. All code used in this study are available on https://github.com/Jingyangkit/SABRE_workspace_GUI.
