# Supplementary material for: Development of a fully automated workstation for conducting routine SABRE hyperpolarization
Source: Sci Rep. 2024 Sep 9;14:21022. doi: 10.1038/s41598-024-71354-x (PMC11384770; doi:10.1038/s41598-024-71354-x)
Supplement: Supplementary file 1 — Supplementary Information. [file 41598_2024_71354_MOESM1_ESM.pdf]

Supporting Information

Development of a fully automated workstation for conducting routine SABRE hyperpolarization

Jing Yang[1], Ruodong Xin[1], Sören Lehmkuhl[1], Jan G. Korvink[1], and Jürgen J. Brandner[1,2]

[1]Karlsruhe Institute of Technology (KIT), Institute of Microstructure Technology (IMT),

[1]Eggenstein-Leopoldshafen, 76344, Germany

[2]Karlsruhe Nano Micro Facility (KNMFi), Hermann-von-Helmholtz-Platz 1,

[2]Eggenstein-Leopoldshafen, 76344, Germany

juergen.brandner@kit.edu

Contents

PTF generator for ^{13}C SABRE-SHEATH **1**

The Magnetic Flux Density inside the Solenoid Coil 3

Calculating the Inductance of the Solenoid Coil 3

Controlling the Direction of PTF by H-Bridge 5

Selecting the appropriate serial resistor to generate μT -field 6

Calculating the Electromagnetic Coil's Time Constant τ_{coil} 7

Averaging the Hamiltonian in situation of shaped magnetic field 8

Validating ^{13}C pyruvate SABRE-SHEATH at the magnetic field generated by PWM **10**

PTF generator for ^{13}C SABRE-SHEATH

In this work, two self-wound solenoid coil A and B are applied for ^{1}H SABRE and ^{13}C SABRE-SHEATH experiments respectively (Figure S1). The ^{1}H SABRE experiments were designed to demonstrate high reproducibility and were conducted using a Polarization Transfer Field (PTF) of 6.5 mT. This magnetic field was generated by solenoid coil A powered by direct current (DC). For ^{13}C SABRE-SHEATH experiments, which necessitated a series of magnetic field sweeps of the PTF, we utilized a pulse-width modulation (PWM) function. This function enabled the generation of a square wave signal through an H-bridge, which was subsequently applied to solenoid coil B. By setting the duty cycle of the PWM and programming the H-bridge

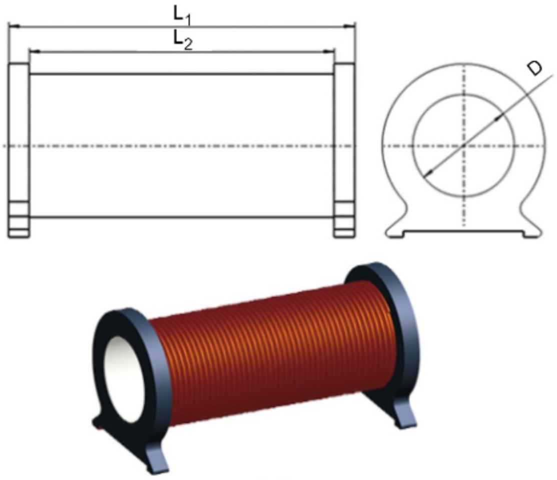

(a)

Parameters	Solenoid coil A	Solenoid coil B
L_1 (cm)	21	21
L_2 (cm)	20	20
D (cm)	4	4
N (Turns)	3500	1300
R_0 (Ω)	50	46.1
PTF range	-10.5 mT- 10.5 mT	-13.5 μT – 13.5 μT

(b)

Figure S1: (a) Schematic illustration of self-wound solenoid coil. (b) Parameters of solenoid coil A and B utilized for generating PTFs in ^{1}H SABRE and ^{13}C SABRE-SHEATH respectively.

circuit, we can automatically control the magnetic fields required for our experiments. The calculation and measurement of the time constant τ_{coil} involve assessing the coil's inductance and resistance, which determine how swiftly it can reach its full magnetic field strength upon signal application and decay once the signal ceases.

The effectiveness of this setup hinges on understanding (1) the time constant τ_{coil} of solenoid coil B, which influences how quickly the coil can respond to changes in the PWM signal; (2) the averaging of Hamiltonian of a square wave-shaped magnetic field with fast period T.

In the following content, the calculation and measurement of the time constant of solenoid coil B are depicted, along with an explanation of how the generation of the PTF is controlled by the PWM signal. Furthermore, the hyperpolarization signals from ^{13}C SABRE-SHEATH at the PTF of -0.6 μT, generated by PWM signal and DC power, are presented to validate that there is a negligible difference in the hyperpolarization effects. This holds true whether employing a fast-period, square wave-shaped magnetic field or a static magnetic field, as long as the average field strengths are equivalent.

The Magnetic Flux Density inside the Solenoid Coil

The relationship between the magnitude of the current applied in the solenoid coil and the strength of the generated magnetic field is described as follows:

$$H \cdot L = N \cdot I \tag{1}$$

Where:

- H is the magnetic field strength measured in Ampere per meter (A/m);
- N is the number of turns in the coil;
- I is the current measured in Ampere (A);
- L is the length of solenoid measured in meters (m).

The magnetic field strength inside the solenoid coil is:

$$B = \mu \cdot H \tag{2}$$

Where:

- B is the magnetic flux density (T);
- μ is the magnetic permeability of the material (T·m/A).

The solenoid coil is placed in air, the magnetic permeability of which is usually considered close to that of a vacuum. Therefore, the vacuum permeability $\mu_0 \approx 4\pi \times 10^{-7}$ T·m/A is chosen for the next calculations.

By combining Equation (1) and Equation (2), and incorporating the parameters provided in the Figure S1, the relationship between the applied current and the magnetic field generated inside coil B can be derived as follows:

$$B_B = \frac{N_B \cdot \mu_0}{L_B} \cdot I_B \approx 0.00816 I_B \tag{3}$$

Calculating the Inductance of the Solenoid Coil

During the calculation of the solenoid coil inductance, the selection of the applicable method is crucial for the subsequent analysis of the circuit time constant, especially in scenarios where the magnetic field needs to be switched quickly. The forward and reverse calculations are two common methods, and in the case of manually wound solenoids, it is important to choose the correct calculation method to minimize errors due to irregularities in the structure. [2,3,6]

The forward calculation method directly calculates the inductance value by considering parameters such as the shape, dimensions, number of turns, and permeability of the solenoid

coil. In previous work, many researchers applied this method for calculating inductance values of solenoids of different shapes. [1, 2] This method is theoretically accurate, but in the case of manual winding, the irregularity of the solenoid may lead to complex mathematical modelling, making the calculation cumbersome and prone to introducing errors. [1] The inverse calculation method means that the inductance value can be solved indirectly by measuring some parameters in the circuit, such as the time constant in current decay process. This approach is rooted in the fact that the inertial effects of the inductor result in a non-instantaneous response of the circuit, requiring a finite duration to reach a steady state. This method is more feasible in practical applications because it can overcome irregularities in the shape of the solenoid coil during manual winding. A variety of inverse calculation methods were summarized in previous work. [5, 7] In the experiments, the method of measuring the time constant in the discharge series RL loop has been chosen, as shown in Figure S2.

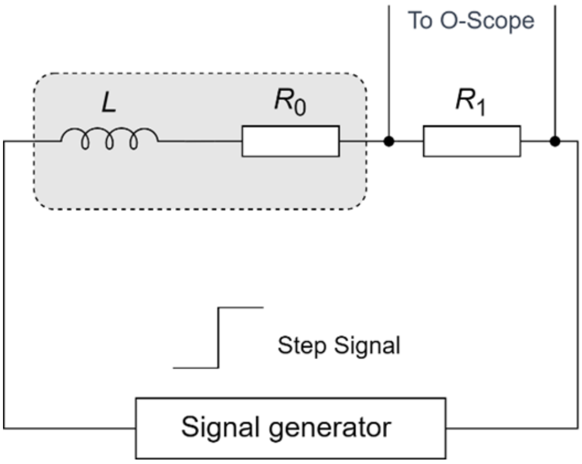

Figure S2: A circuit of series RL loop for measuring the inductance of the self-wound solenoid coil.

In Figure S2, L represents the solenoid coil, R_0 is the resistance of the solenoid coil itself, and R_1 represents the series resistance. The process for measuring inductance involves the following steps. Firstly, the circuit parameters need to be established: The resistance of R_0 is 46.1 Ω and a resistance of 1333 Ω was selected for R_1. Second, energize the circuit and then use an oscilloscope (KEYSIGHT DSOX1240A) to measure the voltage across the resistor R_1. By employing Kirchhoff's laws under the influence of a step voltage (illustrated in Figure S2), the relationship between current i and time t in the RL circuit is depicted as follows:

$$i(t) = \frac{V_{high}}{R_0 + R_1}\left(1 - e^{-\frac{R_0+R_1}{L}t}\right) \tag{4}$$

This implies that after the step voltage occurs, $i(t)$ undergoes an exponential growth process, gradually reaching a steady-state value. Setting $I_0 = \frac{V_{high}}{R_0+R_1}$ and $\tau = \frac{L}{R_0+R_1}$ yields:

$$i(t) = I_0\left(1 - e^{-\frac{t}{\tau}}\right) \tag{5}$$

When $t = \tau$, it gives:

$$i(\tau) = I_0 \left(1 - e^{-1}\right) = 0.63 I_0 \tag{6}$$

where τ represents the time required for the current to increase from 0 to 63% of its steady-state value. In electronics, τ is referred to as the time constant.

From the voltage waveform over time depicted in Figure S3, it is discernible that the time constant τ of the circuit with series of the electromagnetic coil B and the resistor R_1 is approximately 10 μs.

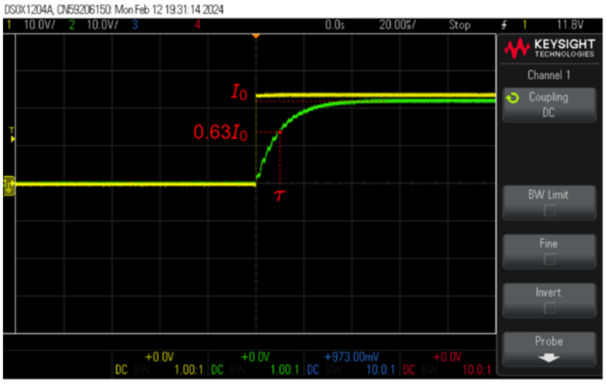

Figure S3: Step signal (yellow line) with voltage across the resistor (green line).

By utilizing the relationship $\tau = \frac{L}{R_0+R_1}$, the inductance value L of the coil is calculated as following:

$$L = \tau \cdot (R_0 + R_1) \approx 13.8 \text{ mH} \tag{7}$$

It needs to emphasize that due to the use of manually wound coils and associated measurement uncertainties, the purposes of Equation (3) and (7) are to provide a qualitative understanding and analysis of the system behavior rather than obtain absolute accurate results.

Controlling the Direction of PTF by H-Bridge

In the PTF generator, the manipulation of the magnetic field direction is facilitated by an H-bridge-based drive circuit. Named for its schematic representation that resembles the letter "H," as depicted in Figure S4. H-bridge is an electronic circuit that allows for the reversal of the polarity of a voltage applied to a load. The components Q_1, Q_2, Q_3, Q_4 are Metal Oxide Semiconductor Field Effect Transistors (MOSFETs), which serve as electronic switches. D_1, D_2, D_3, D_4 are diodes designed to protect the electronic switches. When integrated with PWM, precise adjustments of the voltage at both ends of the load are made achievable. The power supply voltage of the controlling system, represented by V_{DD}, is set at 24 V DC.

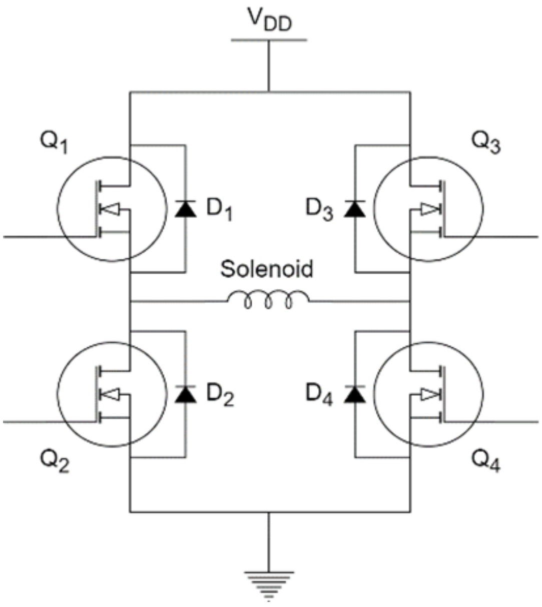

Figure S4: The circuit of H-bridge for switching the polarity of the voltage applied to the solenoid.

Selecting the appropriate serial resistor to generate μT -field

In ^{13}C SABRE-SHEATH procedures, the required magnetic field range is up to 13 μT. According to Equation (3), this necessitates a current range from 0 to 1.59 mA. Given the solenoid coil's resistance R_0 of 46.1 Ω, the required current range translates into a voltage variation from 0 to 0.073 V for the field strength from near-zero to 13 μT. Achieving such voltage control with an accuracy of 0.0056 V per 1 μT present challenges in practice. For the convenience of voltage control within the circuit, a resistor, labeled R_2, is incorporated in series (Figure S5).

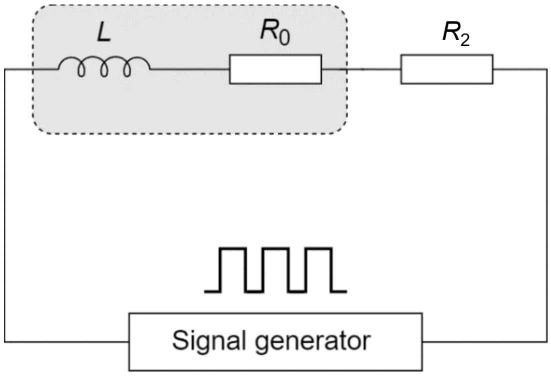

Figure S5: The circuit of the solenoid coil in series with the capacitor R_2 for generating μT-field.

Another factor that needs to be considered when selecting the resistance in the circuit is the relationship between the resistor value and the time constant, which will be discussed in detail in the next section.

Calculating the Electromagnetic Coil's Time Constant τ_{coil}

The time constant in circuits is crucial for characterizing the response capability of the circuit. It quantitatively describes how quickly a circuit can respond to changes in input, fundamentally defining the dynamics of its electrical behavior. The time constant is closely related to the process in which the current changes from its initial variation to a stable state. A small time constant means that the circuit can quickly adapt to changes in signals, achieving the purpose of rapid magnetic field switching. Conversely, a large time constant means that the circuit cannot adapt quickly to signal changes, requiring a longer time for the magnetic field intensity to stabilize after the switching signal occurs. In the RL circuit, where the solenoid coil is connected in series with resistor R_2, the time constant is defined as follows:

$$\tau_{coil} = \frac{L}{R} = \frac{L}{R_0 + R_2} \tag{8}$$

where L is the inductance of the coil and R is the total resistance in the circuit. In case of fixed value of inductance (L), only the effect of the series resistance value (R_2) on the time constant needs to be considered. Both the phase difference and time constant are important physical parameters with significant implications for the functionality of the PTF generator. Therefore, it is necessary to select the circuit parameters (such as the resistance value and the signal frequency) appropriately to ensure that the phase difference and the time constant are within suitable ranges.

From Equation (8), it can be inferred that an increase in the series resistor value leads to a decrease in the time constant. For ^{13}C SABRE-SHEATH at μT-range, an series resistor R_2 with resistance of $8116\ \Omega$ was selected to meet the requirements for both the range and fast response of the magnetic field generated by solenoid coil B. Therefore, a time constant of $1.7\ \mu$s was determined by utilizing Equation (8). Figure S6 depicts the waveform obtained by measurements on oscilloscope with $\tau_{coil} = 1.7\ \mu$s, states the agreement between experimental observations and theoretical predictions.

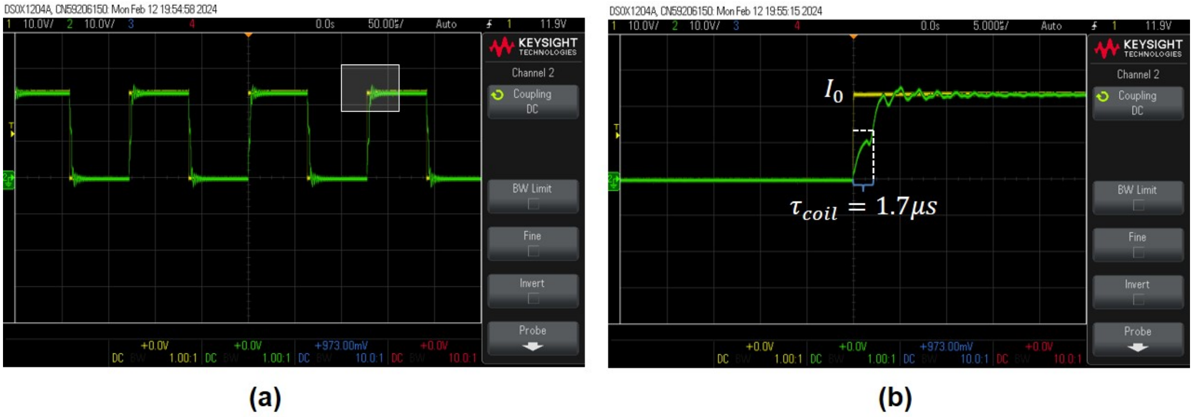

Figure S6: (a) Square wave signal (yellow line) with voltage across the resistor (green line). (b) Local scaling of the signal at the step.

Averaging the Hamiltonian in situation of shaped magnetic field

The Hamiltonian of the spin system at this squre wave-shaped magnetic field $B(t)$ is:

$$\hat{\mathcal{H}}(t) = \Delta\gamma_{CH} B(t)\hat{S}_z + 2\pi\left(J_{HH}\hat{I}_1 \cdot \hat{I}_2 + J_{CH}\hat{I}_1 \cdot \hat{S}\right) \tag{9}$$

where $B(t)$ consists of two magnetic fields $B_h(t)$ and $B_l(t)$, the above equation can be rewritten in:

$$\hat{\mathcal{H}}(t) = \Delta\gamma_{CH} B_h(t)\hat{S}_z + \Delta\gamma_{CH} B_l(t)\hat{S}_z + 2\pi\left(J_{HH}\hat{I}_1 \cdot \hat{I}_2 + J_{CH}\hat{I}_1 \cdot \hat{S}\right) \tag{10}$$

where the magnitude of $B_h = 13.5\ \mu$T and $B_l = 0$ are corresponding to the high and low level of the PWM signal. The working times of B_h and B_l in a period are t_1 and t_2 respectively (Figure S7). This gives:

$$\hat{\mathcal{H}}(t) = \Delta\gamma_{CH} B_h(t_1)\hat{S}_z + 2\pi\left(J_{HH}\hat{I}_1 \cdot \hat{I}_2 + J_{CH}\hat{I}_1 \cdot \hat{S}\right) \tag{11}$$

$$= \hat{\mathcal{H}}_1(t) + 2\pi\left(J_{HH}\hat{I}_1 \cdot \hat{I}_2 + J_{CH}\hat{I}_1 \cdot \hat{S}\right) \tag{12}$$

When the period of the pulse shaped magnetic field T is faster than any other term in the Hamiltonian at any time, the average Hamiltonian can be applied. [4] The term $\hat{\mathcal{H}}_1(t)$ can be averaged and this gives:

$$\overline{\hat{\mathcal{H}}}_1^{(0)} = \frac{1}{T}\int_0^{t_1} \hat{\mathcal{H}}_1(t)\, dt \tag{13}$$

$$= \frac{t_1}{T}\Delta\gamma_{CH} B_h \hat{S}_z \tag{14}$$

where the ratio $\frac{t_1}{T}$ equals to the duty cycle of the PWM signal applied on the electromagnetic coil. By the definition of the duty cycle of PWM function, the average voltage is:

$$\overline{V} = \frac{t_1}{T} V_h \tag{15}$$

For an electromagnetic coil, the generated magnetic field strength is proportional to the applied voltage ($B \propto V$). The relationship of the average magnetic field $\overline{B}$ and the high magnetic field B_h gives:

$$\overline{B} = \frac{t_1}{T} B_h \tag{16}$$

Therefore, Equation 14 can be rewritten in:

$$\overline{\hat{\mathcal{H}}}_1^{(0)} = \Delta\gamma_{CH} \overline{B} \hat{S}_z \tag{17}$$

and the total Hamiltonian is:

$$\hat{\mathcal{H}}(t) = \Delta\gamma_{CH}\overline{B}\hat{S}_z + 2\pi\left(J_{HH}\hat{I}_1 \cdot \hat{I}_2 + J_{CH}\hat{I}_1 \cdot \hat{S}\right) \tag{18}$$

Hence, when the spin system evolves under square-wave shaped magnetic field with period faster than the coherence dynamics of spin evolution, there is negligible difference in hyperpolarization outcomes generated whether at a static magnetic field or at instantaneously changing field, as long as the average magnetic field strength is equivalent to that of the static field.

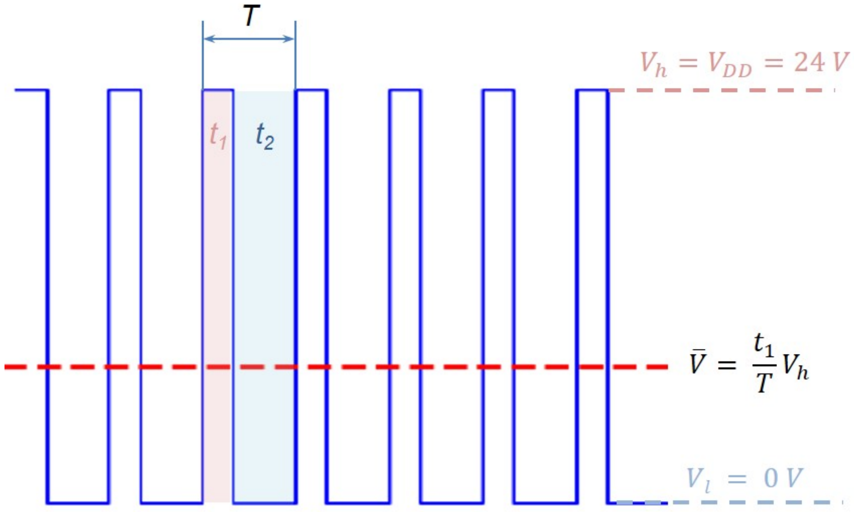

Figure S7: Schematic illustration of the average voltage achieved by PWM signal with corresponding duty cycle. Within the period time T, the working durations of high and the low level are t_1 and t_2 respectively. The duty cycle $\frac{t_1}{T}$ is the ratio between the high level duration and the period time. The average voltage over time can be controller by adjusting the duty cycle.

In this work, the PWM signal with the default frequency on Arduino $f = 976.56$ Hz, was employed in the experimental procedures of field sweeping for determining the optimal PTF, buildup time measurement, and T_1 relaxation time measurement for generating desired magnetic field. The time constant of the electromagnetic coil $\tau_{coil} = 1.7\ \mu$s has been calculated theoretically and measured experimentally. With τ_{coil} is much small than the period time of PWM ($T = \frac{1}{f} = 1024\ \mu$s), the resulting magnetic field closely follows the square wave pattern dictated by the PWM signal. The period time $T \ll (\Delta\gamma_{CH}B_h)^{-1} = 2324\ \mu$s, the output of the hyperpolarization under a rapidly changing square wave-shaped magnetic field is nearly identical to that observed under a static magnetic field with strength equal to $\overline{B}$. Therefore, by manipulating the duty cycle of the PWM signal of the PTF generator, we were able to control the hyperpolarization at desired magnetic field.

Validating ^{13}C pyruvate SABRE-SHEATH at the magnetic field generated by PWM

To demonstrate the the average effect of hyperpolarization at square wave-shaped magnetic field with fast period, serial experiments of ^{13}C pyruvate hyperpolarization at its optimal PTF ($B_{PTF} = -0.6$ μT) generated by the PWM with period of 1024 μs and by the DC power were conducted. For the DC power generated magnetic field, we chose a 12V DC power and manually adjusted the current to achieve the static field of -0.6 μT.

For each magnetic generation approach, three times hyperpolarization of the same sample solution were conducted sequentially. The results are shown in Figure S8, the spectra with blue and red color are the signal of ^{13}C pyruvate signal hyperpolarized at -0.6 μT generated by PWM function and DC power respectively. By comparison the spectra, the signal intensities of the free and bound pyruvate are nearly identical under both conditions. This demonstrates that the magnetic field generated by the PWM approach with a frequency of 976.56 Hz can effectively be treated as a static magnetic field for the purposes of our experiments.

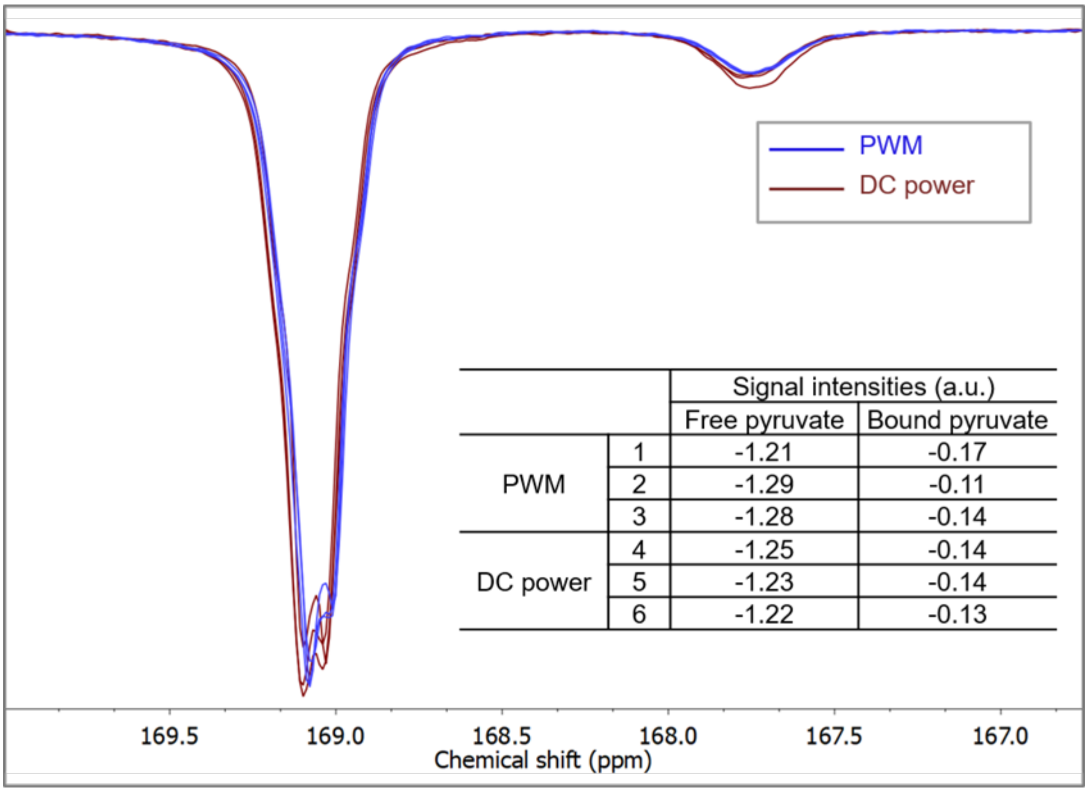

Figure S8: Spectra of ^{13}C pyruvate hyperpolarized at -0.6 μT generated by PWM (blue) and by DC power (red).

References

[1] HA Aebischer. Inductance formula for rectangular planar spiral inductors with rectangular conductor cross section. *Advanced electromagnetics*, 9(1):1–18, 2020.

[2] Andreia Faria, Luís Marques, Carlos Ferreira, Filipe Alves, and Jorge Cabral. A fast and precise tool for multi-layer planar coil self-inductance calculation. *Sensors*, 21(14):4864, 2021.

[3] David W Knight. Solenoid inductance calculation. *DW Knight*, 2013.

[4] Jacob R Lindale. *Understanding and Optimizing Dynamics in Hyperpolarized Magnetic Resonance*. PhD thesis, Duke University, 2021.

[5] Se-Yuen Mak. Six ways to measure inductance. *Physics education*, 37(5):439, 2002.

[6] Christian Peters and Yiannos Manoli. Inductance calculation of planar multi-layer and multi-wire coils: An analytical approach. *Sensors and Actuators A: Physical*, 145:394–404, 2008.

[7] Yas Hosseini Tehrani and Seyed Mojtaba Atarodi. An efficient solution to measure inductance on-the-fly. *Measurement*, 223:113670, 2023.
